# Supplementary figures and images for: Transcriptome-wide identification of novel circular RNAs in soybean in response to low-phosphorus stress
Source: PLoS One. 2020 Jan 21;15(1):e0227243. doi: 10.1371/journal.pone.0227243 (PMC6974154; doi:10.1371/journal.pone.0227243)

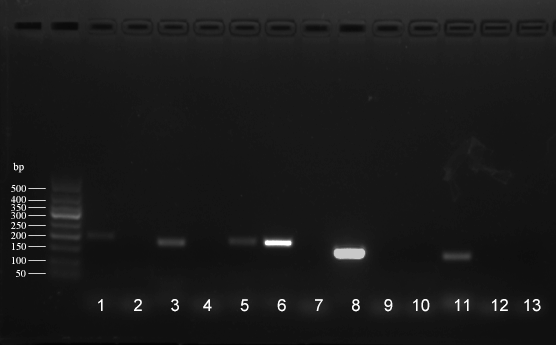

Supplement: S2 Fig — Thirteen circRNAs are shown in the figure. The successfully validated circRNAs included the following: 1, novel_circ_000274; 3, novel_circ_000338; 5, novel_circ_000035; 6, novel_circ_000093; 8, novel_circ_000108; and 11, novel_circ_000237. The unsuccessfully validated circRNAs included the following: 2, 4, 7, 9, 10, 12 and 13 (M, DL500 marker). Detailed information can be found in S3 Table. (TIF) [file pone.0227243.s002.tif]
