# Supplementary material for: Transcriptome-wide identification of novel circular RNAs in soybean in response to low-phosphorus stress
Source: PLoS One. 2020 Jan 21;15(1):e0227243. doi: 10.1371/journal.pone.0227243 (PMC6974154; doi:10.1371/journal.pone.0227243)
Supplement: Attachment — Submitted filename: Review report.docx [file pone.0227243.s012.docx]

In this study, authors performed the genome-wide identification of circular RNAs in soybean, and then profiling their expression patterns, detecting the differentially expressed ones, carried out GO annotation and enrichment, and finally experimentally confirmed selected ones. It is meaningful and interesting to readers. Generally speaking, the work was well designed and the result was sufficient to support their conclusion. Meanwhile, the writing and description are OK. However, I have a few comments that should be addressed to ensure the improving of the manuscript.

**Major points:**

The paper of Zhao et al. 2017. Scientific report. also reported the circRNAs in soybean, which should be cited and discussed by authors. The previously results reported by Zhao et al (2017) and the results reported by authors should be compared and discussed, including the number of detected circRNAs, the same and novel circRNAs between the two studies, the circRNAs identification procedures. Because these are important to fully understand the circRNAs reported by authors in this paper. Moreover, several recently published article related to circRNAs in plants under abiotic stress including cucumber should be mentioned and maybe discussed in the article (Zhu et al 2019, BMC, Identification of cucumber circular RNAs responsive to salt stress).

L159. Previously,	gene was defined as DNA fragments coding proteins. With the advance of knowledge, besides coding genes, numerous non-coding genes, including miRNA, lncRNA, circRNA and so on, were reported. It is similar in soybean. I suggest authors carefully used the word “source gene”, as from your description, it apparently only represents the coding genes. Meanwhile, I suggest that authors should analysis which circRNA origin from lncRNA to further expand the source genomic region of circRNA.

L151. It is OK that the circRNA “were annotated by blasted against the circBase”. But the parameters were not given. Furthermore, how to make sure those annotated circRNA are not novel circRNAs, as those well-matched hits also can be different circRNAs, although they shared same annotation based on BLAST analysis.

L228. Authors said that “indicating that the circRNAs were generated by various mechanisms”. It is much unclear. Short and long circRNAs and uneven distribution are the result of which kind of mechanisms? Please clarify.

**Minor points:**

L38. Change the keywords, as some of them had appeared in the title.

L55. Change “is” to “are”.

L77. Change “or” to “and”.

L110. It should be Orthographic, not Italic, in “µM”. Change thoroughly.

L111. In Hoagland's, it should be Hoagland’s.

L115. Missing blank between 80 and °C. Please check thoroughly to avoid the missing blank between number and unit.

L135. Change “200-500” to “200 to 500”.

L141. Remove the “TM” in HiSeqTM 4000, or make it superscript.

L146. Rewrite the sentence “The reads were filtered were based on three rules”.

L148. Missing blank before and after “≤”.

L146-148. As words after (1) and (2) are complete sentence, the (3) also should be complete sentence to match the same pattern.

L174. Change the form of “2(^-△△CT^)”. Usually, it shows as 2^-△△Ct^.

L149 and L197. Keep the same format of “Wm82.a2.v1”. In L149, it is Italic; in L197, it is not.

L214. Remove “an”. As it is one to more, not always one exon.

L287. Make sure there is blank before and after “>” and thoroughly check all over the paper.

L343. The “cis” should be Italic.

L379. Add “” to the word negative to keep the same pattern as “positive”.

L381. As you divided circRNAs into “positive” and “negative”. Please show how many circRNAs are “positive” and how many are “negative” in the most circRNAs (88%).

L401. Dose these 570 miRNAs belong to soybean. If it is, where and how did you collect them. Please clarify.

L404. Please give several examples of “specific-expression novel DE circRNAs” and show circRNAs target to which miRNA.

L418-L419. Please convince me that the 570 miRNAs were only targeting to 95 mRNAs.
